# Case report: Successful treatment of non-bullous lichen planus pemphigoides with dupilumab

**DOI:** 10.3389/fmed.2022.1023458

**Published:** 2022-12-16

**Authors:** Si-Zhe Li, Ying-Han Xie, Si-Hang Wang, Rou-Yu Fang, Hong-Zhong Jin, Ya-Gang Zuo

**Affiliations:** Department of Dermatology, State Key Laboratory of Complex Severe and Rare Diseases, Peking Union Medical College Hospital, Chinese Academy of Medical Science and Peking Union Medical College, National Clinical Research Center for Dermatologic and Immunologic Diseases, Beijing, China

**Keywords:** lichen planus pemphigoids, non-bullous, dupilumab, vaccine, lichen planus, corticosteroid

## Abstract

Lichen planus pemphigoides (LPP) is a rare autoimmune bullous disease, characterized by the coexistence of lichen planus and subepidermal bullae. However, the minority of LPP patients present with papules rather than vesicles or blisters, which is defined as non-bullous LPP. The diagnosis of LPP relies on manifestations, histopathology, serological assay, and direct immunofluorescence of linear disposition of IgG and/or C3 at the basement membrane zone. Up to now, no standard therapeutic strategies have been proposed for the treatment of LPP. Herein, we describe an uncommon non-bullous LPP patient with widespread papules and erythema, probably induced by vaccination. During hospitalization, he had a poor response to the conventional treatment of topical and systemic corticosteroids, and his condition was finally alleviated by the addition of dupilumab. For LPP patients with a traditional medication failure, or who were not suitable for a higher dose of corticosteroids, a combination with dupilumab could be an alternative option.

## Introduction

Lichen planus pemphigoides (LPP) is an uncommon subtype of autoimmune bullous disease (AIBD). According to a systemic review published recently, its prevalence is estimated to be about 1 per 1,000,000 patients (1). This disease is more likely to affect patients in their third to sixth decades with a slightly female predominance (2, 3). Typically, LPP is characterized by the coexistence of both lichen planus (LP) and bullae. However, a minority of LPP patients manifest with papules rather than blisters, which is referred to as non-bullous LPP (4, 5). The diagnosis of LPP relies on the clinical feature, biopsy, and immunopathological characteristics. In LPP, bullae could arise in previously normal skin. Whereas in bullous lichen planus (BLP), another subtype of LP with blister, bullae always develop within pre-existing lesions of LP. Direct immunofluorescence (DIF) of perilesional biopsy is indispensable for the differential diagnosis of LPP and BLP. More specifically, a linear disposition pattern of IgG and/or C3 in the basement membrane zone (BMZ) could be detected in LPP patients rather than BLP ones (6). It was declared that medication, infections, vaccines, and tumors could be the associated triggers of LPP (1). Only two cases have been illustrated to be vaccine-related: one was triggered by hepatitis A vaccination (7) and the other by non-avalent human papillomavirus vaccination (8). No such cases following COVID-19 vaccination have been recorded till now. So far, no standard treatment strategies for LPP have been defined. Based on limited clinical experience, systemic corticosteroids are the most frequently prescribed agents. Dapsone, acitretin, and topical corticosteroids could serve as alternatives when systemic corticosteroids were not available (1). Herein, we reported a non-bullous LPP patient who was probably triggered by COVID-19 vaccination and successfully treated by dupilumab and systemic corticosteroid.

## Case description

**Figure 1:**
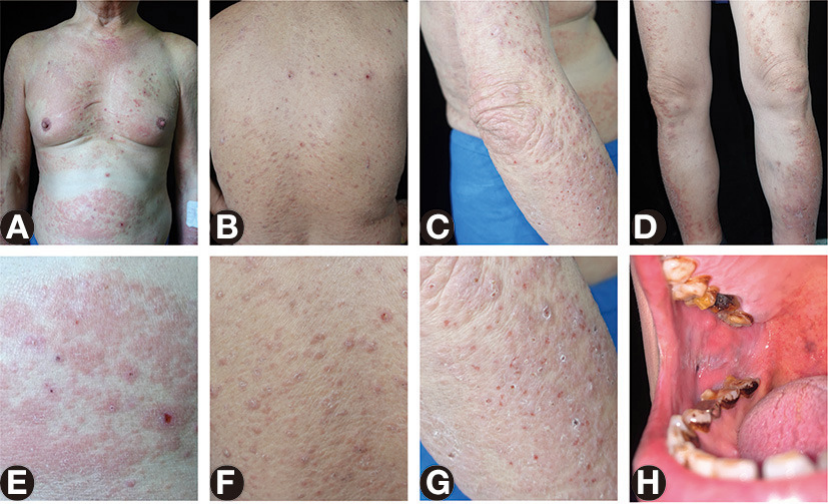
Skin and oral lesions of the patient. **(A–D)** Widespread red papules, edematous erythema, erosions, and multiple crusts on the neck, trunk, and extremities. **(E–G)** Close-up view of the abdomen, back, and arm. **(H)** Violet plaques with white stripes on the right buccal mucosa.

A 69-year-old man presented to our clinic with a 1-year history of red papules and plaques with severe itching. He had a 20-year history of oral LP. One year previously, skin lesions started 3 days after the reception of the second dose of COVID-19 vaccination, which firstly manifested with isolated papules on both lower extremities and then quickly coalesced and spread throughout the entire body. Prior to the presentation, he had been treated with compound glycyrrhizin, antihistamine agent, tripterysium glycosides, fluticasone propionate, and fluoconodimethyl sulfoxide, resulting in an unsatisfactory response. Physical examination revealed widespread red papules, edematous erythema, and multiple crusts on the scalp, face, neck, trunk, and extremities (Figures 1A–G). Violet plaques with white stripes were observed on the bilateral buccal mucosa and soft palate (Figure 1H). There were no nail or scalp lesions. The laboratory investigation showed eosinophilia (eosinophil percentage, 11.4%) and an elevated serum total IgE production (150 kU/L, normal range, 0–60 kU/L). IgG autoantibody targeting NC16A domain of bullous pemphigoid (BP) 180 by enzyme linked immunosorbent assay (ELISA) was weakly positive (12 U/ml, normal range, <9 U/ml).

**Figure 2:**
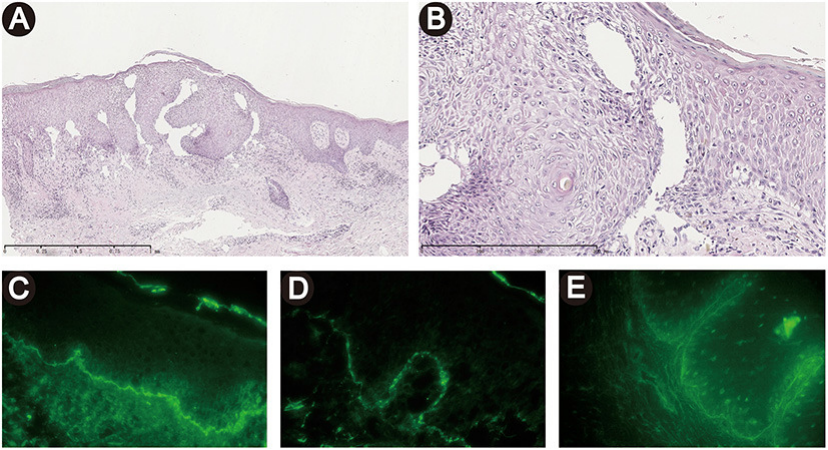
Histopathological and immunofluorescent findings. **(A, B)** Histopathology demonstrated intercellular epidermal edema, a subepidermal blister with an underlying sparse dermal perivascular infiltrate of lymphocytes and eosinophils. [H&E staining, original magnification, × 50 **(A)**, × 200 **(B)**]. **(C, D)** Direct immunofluorescence showed linear depositions of IgG **(A)** and C3 **(B)** at the basement membrane zone. **(E)** Indirect immunofluorescence utilizing monkey esophagus substrate demonstrated a linear deposition of serum IgG along the basement membrane zone.

Histopathology demonstrated intercellular epidermal edema, a subepidermal blister with an underlying sparse dermal perivascular infiltrate consisting of lymphocytes and eosinophils (Figures 2A,B). DIF showed linear depositions of IgG and complement component 3 at the BMZ (Figures 2C,D). Indirect immunofluorescence utilizing monkey esophagus substrate demonstrated serum anti-BMZ autoantibodies of IgG at 1:40 dilution (Figure 2E). Other medical history included hypertension (well-controlled by nifedipine, bisoprolol fumarate, and losartan potassium tablets for about 20 years) and diabetes mellitus type 2 (poorly controlled by acarbose and isophane protamine biosynthetic human insulin, with a fast blood glucose of 9.8 mmol/L). Based on the clinical features, histopathological and immunofluorescent findings, as well as an ELISA result, a diagnosis of BP was made at first, with an evaluation of BP disease area index (BPDAI) score of 104.

**Figure 3:**
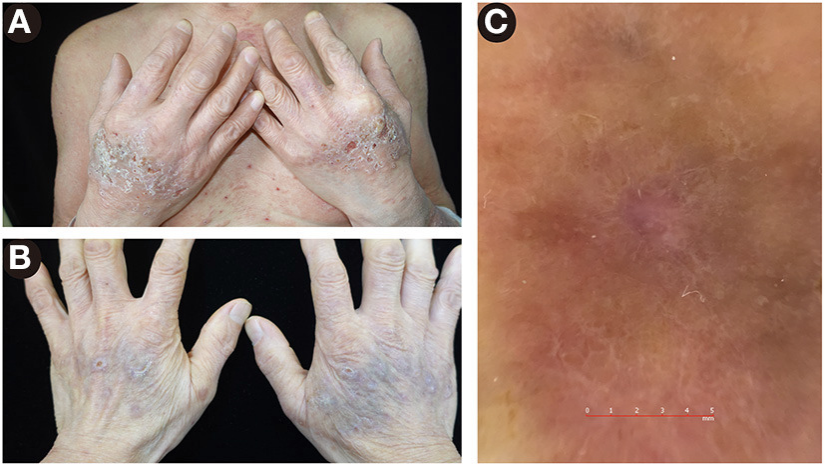
Lesions on the dorsal aspects of the hands. **(A)** The edematous erythema and crusts before therapy. **(B)** The violaceous flat-topped papules and plaques after therapy. **(C)** By dermatoscope, Wickham's striae were presented, a feature of lichen planus.

At first, a conventional therapy strategy of prednisolone 50 mg/d intravenously and halometasone triclosan ointment topically was prescribed. Four days later, a limited improvement was observed. Taking his medical history of extensive mucocutaneous involvement, hypertension, diabetes mellitus, and poor response to previous treatments into consideration, an additional agent of dupilumab 600 mg subcutaneously was introduced, resulting in a rapid resolution of skin lesions in 3 days. After one week, the dose of dupilumab reduced to 300 mg subcutaneously every other week, and prednisolone was gradually tapered to 30 mg per day, with a re-evaluated BPDAI score of 23. More interestingly, the edematous erythema and crusts on the dorsal aspects of the hands (Figure 3A) improved after therapy and left some violaceous flat-topped papules and plaques (Figure 3B). Wickham's striae were observed on those papules and plaques by dermatoscope (Figure 3C), which were the hallmark of LP. Ultimately, non-bullous LPP was diagnosed.

**Figure 4:**
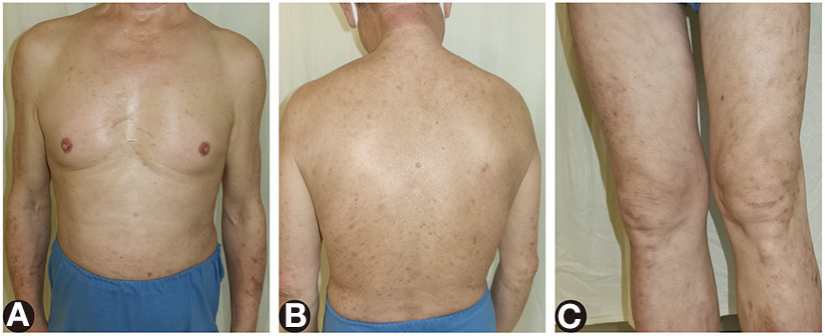
The remaining lesions after treatment of dupilumab and systemic corticosteroids for 2 months. **(A–C)** Most of the red papules, edematous erythema, and erosions cleared and left pigmentation.

Two months later, oral involvement and 90% of the skin lesions were cleared off, and the eosinophil percentage and serum anti-BP180NC16A IgG level both returned to normal on dupilumab 300 mg subcutaneously every other week, methylprednisolone 12 mg per day, and topical halometasone triclosan ointment (Figures 4A–C).

## Discussion

LPP is a rare autoimmune dermatosis, of which the hallmark is the coexistence of both LP and blisters. Distinct from BLP, blisters of LPP can be found on normal skin which has not been previously affected by LP. However, for certain patients with atypical lesions, it remains a challenge to differentiate LPP from BLP cases only on clinical manifestations. Further dermatology examinations like histopathology, serological assays, and DIF are required. For BLP patients, it is hypothesized that the development of vesicles and bullae derived from extensive epidermal inflammatory infiltrations rather than erroneous overexpression of antibodies targeting structural proteins at the BMZ (6). Consequently, blisters of BLP under the microscope would present with vacuolar changes within the basal cell layer. Moreover, the result of DIF and the serological assays of IgG targeting BP180 in BLP patients would be negative, which could serve as strong evidence for the differential diagnosis between BLP and LPP. In this case, the patient was firstly diagnosed as BP, but the LP lesions on the dorsal aspects of the hands were subsequently observed and confirmed by dermoscope during his hospitalization. Moreover, the papules arose outside of LP lesions, and immunofluorescent findings were presented, supporting the diagnosis of LPP. In addition, linear deposits of auto-antibodies or complements by DIF could also be present in several autoimmune connective tissue disorders, such as systemic lupus erythematosus. However, lesional and histopathological findings of those diseases are characteristics, and other organs are also affected, rarely presenting with dermal-epidermal separation.

A few cases of LPP could present without vesicles or bullae and are defined as non-bullous LPP. So far, only two such cases have been reported (4, 5). Both seemed to be induced by the administration of new drugs and the lesions were mainly localized on the lower limbs. Diagnosis of these patients remains a challenge. One of them was finally confirmed by histopathology (5) and the other by a linear disposition of IgA along the BMZ of perilesional skin by DIF assay (4). Similarly to the previous case reports, this patient did not present with typical blisters, making the initial diagnosis at his first visit difficult. However, the recent eruption of mucocutaneous lesions in the patient were not drug-related and presented with a whole-body involvement. As for the oral lesions, although the patient reported a 20 year medical history of both the oral LP and the administration of losartan potassium, it was difficult for him to retrace which one was more previous and evaluate whether this lesion was drug-related. The diagnosis was ultimately confirmed by the positive serological detection of IgG targeting at BP180 NC16A, the typical dermoscopic features, and the DIF deposition pattern.

The pathogenetic mechanism of LPP remains a mystery. It is hypothesized that the chronic inflammatory status of skin, resulting in an exposure of hidden type XVII collagen antigens in the BMZ, finally induced an excessive autoimmune reaction targeting the BMZ (9). In addition, LPP has been reported to be related with multiple factors (1), including drugs (angiotensin converting enzyme inhibitor, PD-1 inhibitor, antidepressants, etc.), infections (varicella and hepatitis B), UV therapy (narrow band UVB and psoralen UVA therapy), vaccinations (7, 8), tumors, and body tattooing (10). For the two cases triggered by vaccines, one was induced by hepatitis A vaccination (9) and the other by non-avalent human papillomavirus vaccination (8). The administration of COVID-19 vaccination could induce various cutaneous adverse effects, including AIBDs. Elena *et al*. reviewed all the reported AIBD cases triggered by COVID-19 vaccination, including BP (74.3%), pemphigus vulgaris (17.1%), linear IgA bullous dermatosis (5.7%), and pemphigus foliaceus (2.9%) (11). Cross-reaction due to molecular mimicry mechanism could be the most accredited pathogenetic hypothesis in the patients induced by vaccine (12). To our knowledge, this is the first case of LPP probably induced by COVID-19 vaccine. Our patient had suffered from oral LP for more than 20 years before this eruption, which accounted for a long-period exposure of the BMZ antigens, leaving him at much higher risk of autoimmune dysregulation than normal individuals, and finally induced by the administration of the vaccine. The COVID-19 vaccination might be a trigger of pemphigoid. Nevertheless, it cannot be proved. The patient could not remember the details about the COVID-19 vaccination. Apart from two doses of the COVID-19 vaccination, the patient had not received any other vaccinations in recent years.

Due to its rarity, no consensus has been reached for the treatment of LPP. According to the literature research (1), systemic corticosteroids are generally sufficient for the remission of mucocutaneous lesions. Doses prescribed for Japanese patients could be lower (i.e., 15 mg, proved to be effective) than those admitted in other counties (0.5–1 mg/kg body weight). In consideration of the adverse effect of systemic corticosteroids, dapsone, doxycycline, acterin, athioprin, mycophenolate mofetile, and topical administration of corticosteroids could be substitutable choices for the control of disease. As for biologics, limited reports have illustrated that LPP can be alleviated by ustekinumab (13) and tildrakizumab (14), while rituximab (15) seems to be insufficient. Dupilumab has been approved for the administration of asthma and moderate-to-severe atopic dermatitis (16). It is a kind of human monoclonal antibody targeting interleukin-4 receptor α, and regulates type 2 inflammation by blockading interleukin (IL)-4 and IL-13 signaling pathway (17). Recently, two clinical studies have demonstrated the efficacy of dupilumab in BP patients with a poor response to traditional therapy strategies. In those patients, dupilumab can lead to both a more rapid clearance of lesions and accelerate the tapering process of corticosteroids (18, 19). Nevertheless, the efficiency of dupilumab in LPP has not been reported. It was speculated that dysregulation of type 2 inflammation to BP180 NC16A, the target of auto-antibody of LPP, might be necessary for the development of LPP (1). Dupilumab could inhibit the Th2 response through directly blocking IL-4 and IL-13 pathway, and indirectly reducing IgE level and eosinophil activity by inhibiting proliferation of pre-B cell, modifying the expression of B cell, and downregulating the production of Th2-related chemokines (eotaxin, chemokine C-C motif ligand (CCL) 13, and CCL18) (18). In our patient, previous treatment, including immunosuppressive agent and topical corticosteroids, seemed to be insufficient. After administration of corticosteroids, the rash and pruritus partially improved. However, it still bothered the patient, especially at night. Given the multiple complications, it was not a good choice to increase the dose of corticosteroids. Other adjuvant conventional immunosuppressants generally work slowly. Therefore, we prescribed dupilumab to the patient. After that, the rash and pruritus resolved rapidly, and eosinophil percentage and IgG targeting BP180 NC16A returned to normal. Above all, the resolution of the disease attributes to both agents, and dupilumab might be an effective option for LPP, which remains to be verified in future studies.

## Conclusion

In conclusion, we reported a rare non-bullous LPP case. The patient had a long preceding history of oral LP and developed widespread LP and papules 3 days after receiving the second dose of COVID-19 vaccination. It is suspected that consistent exposure of hidden antigens due to lichenoid lesions placed the patient at a high risk of autoimmune dysregulation. After the administration of vaccination, cross-reaction related to mimicry system finally damaged the vulnerable balance of his immune system and induced the eruption of rashes. On medication, he had a poor response to topical or systemic corticosteroids. An attempt of dupilumab combination therapy was well tolerated and led to a rapid alleviation of skin lesions, which may provide a new selection for the treatment of LPP patients who are not suitable for high doses of systemic corticosteroids.

## Data availability statement

The original contributions presented in the study are included in the article/supplementary material, further inquiries can be directed to the corresponding author.

## Ethics statement

The studies involving human participants were reviewed and approved by Peking Union Medical College Hospital, Chinese Academy of Medical Science. The patients/participants provided their written informed consent to participate in this study. Written informed consent was obtained from the individual(s) for the publication of any potentially identifiable images or data included in this article.

## Author contributions

The manuscript was written by S-ZL and Y-HX, and revised by S-ZL, Y-HX, S-HW, R-YF, H-ZJ, and Y-GZ. All authors contributed to the article and approved the submitted version.

## References

[1] Hübner F Langan EA Recke A. Lichen planus pemphigoides: from lichenoid inflammation to autoantibody-mediated blistering. Front Immunol. (2019) 10:1389. 10.3389/fimmu.2019.01389 31312198 PMC6614382

[2] Balighi K Mousavi A Hatami P Daneshpazhooh M Ghiasi M Hesari KK. A 10-year survey on lichen planus pemphigoides in Iran: a therapeutic conundrum. Dermatol Ther. (2022) 35:e15387. 10.1111/dth.15387 35174587

[3] Zaraa I Mahfoudh A Sellami MK Chelly I El Euch D Zitouna M. Lichen planus pemphigoides: four new cases and a review of the literature. Int J Dermatol. (2013) 52:406–12. 10.1111/j.1365-4632.2012.05693.x 23331194

[4] Lamberts A Diercks GFH Pas HH Horváth B. Non-bullous lichen planus pemphigoides: a case report. Acta Derm Venereol. (2020) 100:adv00156. 10.2340/00015555-3523 32424432 PMC9137362

[5] Tan AJ Vaidya S. Non-bullous lichen planus pemphigoides possibly induced by venlafaxine. Australas J Dermatol. (2016) 57:154–5. 10.1111/ajd.12306 27124647

[6] Papara C Danescu S Sitaru C Baican A. Challenges and pitfalls between lichen planus pemphigoides and bullous lichen planus. Australas J Dermatol. (2022) 63:165–71. 10.1111/ajd.13808 35196400

[7] Lahouel M Aounallah A Mokni S Sriha B Belajouza C Denguezli M. Severe childhood lichen planus pemphigoides after hepatitis A vaccination. Skin Health Dis. (2022) 2:e94. 10.1002/ski2.94 35677923 PMC9168010

[8] Pizzatti L Ferreli C Conti B Atzori L Pinna G Pilloni L. Childhood erythrodermic lichen planus pemphigoides after nonavalent human papillomavirus vaccination. JAAD Case Rep. (2020) 6:431–3. 10.1016/j.jdcr.2020.03.008 32382638 PMC7200189

[9] Lodi G Scully C Carrozzo M Griffiths M Sugerman PB Thongprasom K. Current controversies in oral lichen planus: report of an international consensus meeting. Part 1 Viral infections and etiopathogenesis. Oral Surg Oral Med Oral Pathol Oral Radiol Endod. (2005) 100:40–51. 10.1016/j.tripleo.2004.06.077 15953916

[10] Lim A Tang PY Oh CC. Lichen planus pemphigoides after body tattooing. J Cosmet Dermatol. (2020) 19:3048–52. 10.1111/jocd.13553 32542984

[11] Calabria E Canfora F Mascolo M Varricchio S Mignogna MD Adamo D. Autoimmune mucocutaneous blistering diseases after SARS-CoV-2 vaccination: a case report of *Pemphigus vulgaris* and a literature review. Pathol Res Pract. (2022) 232:153834. 10.1016/j.prp.2022.153834 35278817 PMC8896864

[12] Vojdani A Kharrazian D. Potential antigenic cross-reactivity between SARS-CoV-2 and human tissue with a possible link to an increase in autoimmune diseases. Clin Immunol. (2020) 217:108480. 10.1016/j.clim.2020.108480 32461193 PMC7246018

[13] Knisley RR Petropolis AA Mackey VT. Lichen planus pemphigoides treated with ustekinumab. Cutis. (2017) 100:415–8. 29360890

[14] Kerkemeyer KL Pinczewski J Sinclair R. Successful treatment of recalcitrant lichen planus pemphigoides with tildrakizumab. Australas J Dermatol. (2020) 61:e366–e8. 10.1111/ajd.13263 32141608

[15] Schmidgen MI Butsch F Schadmand-Fischer S Steinbrink K Grabbe S Weidenthaler-Barth B. Pembrolizumab-induced lichen planus pemphigoides in a patient with metastatic melanoma. J Dtsch Dermatol Ges. (2017) 15:742–5. 10.1111/ddg.13272 28622432

[16] Bagel J Nguyen TQ Lima H Jain N Pariser DM Hsu S. Baseline demographics and severity and burden of atopic dermatitis in adult patients initiating dupilumab treatment in a real-world registry (PROSE). Dermatol Ther (Heidelb). (2022) 12:1417–30. 10.1007/s13555-022-00742-w 35590038 PMC9209562

[17] Bieber T. Interleukin-13: targeting an underestimated cytokine in atopic dermatitis. Allergy. (2020) 75:54–62. 10.1111/all.13954 31230370

[18] Abdat R Waldman RA de Bedout V Czernik A McLeod M King B. Dupilumab as a novel therapy for bullous pemphigoid: a multicenter case series. J Am Acad Dermatol. (2020) 83:46–52. 10.1016/j.jaad.2020.01.089 32179082

[19] Zhang Y Xu Q Chen L Chen J Zhang J Zou Y. Efficacy and safety of dupilumab in moderate-to-severe bullous pemphigoid. Front Immunol. (2021) 12:738907. 10.3389/fimmu.2021.738907 34721404 PMC8552038

